# Sex and hand differences in haptic processing: implications for mental rotation ability

**DOI:** 10.1186/s13293-025-00693-9

**Published:** 2025-02-03

**Authors:** Daniela E. Aguilar Ramirez, Claudia L. R. Gonzalez

**Affiliations:** Department of Kinesiology and Physical Education, University of Lethbridge, 4401 University Drive, Lethbridge, AB T1K 3M4 Canada

**Keywords:** Haptic processing, Mental rotation, Sex differences, Sensorimotor system, Hemispheric specialization

## Abstract

It has been proposed that the sensorimotor system provides a foundation for the development of cognitive abilities and their hemispheric specialization. In this study, we investigated the potential relationship between haptic processing and mental rotation ability, both of which are typically lateralized to the right hemisphere. Previous research has also indicated that males tend to outperform females in both functions. The current study investigates how the sensorimotor-haptic system relates to mental rotation ability, specifically to examine the influence of hand performance (as a proxy for hemispheric specialization) and biological sex on this relationship. Seventy-five participants (n = 41 females) completed a haptic task, and the well-known mental rotation test (MRT) developed by Shepard and Metzler (Science 171:701–3, 1971). Results confirmed a positive correlation between performance on the haptic and MRT tasks. Further, males outperformed females in both tasks. However, when sex and hand performance were considered, males were better in the haptic task, but only when using their left-hand. Moreover, left-hand haptic performance was the sole predictor of MRT performance. These findings suggest that sex differences in haptic processing may contribute to the observed sex differences in mental rotation ability, supporting the view that sensorimotor processes shape cognitive function and its hemispheric lateralization.

## Plain language summary

The sensorimotor system has been proposed to contribute to cognitive development. The haptic system, a key component of this system, integrates touch and proprioception (awareness of limb positions and movements) to guide actions. Notably, the right hemisphere of the brain, which controls the left hand, is specialized for haptic processing. This hemisphere is also crucial for mental rotation (MR)—the cognitive ability to mentally visualize how objects appear when rotated. Interestingly, males have been found to outperform females in both haptic and MR tasks. This study aimed to explore the relationship between haptic processing and MR ability, particularly examining whether this relationship is more pronounced in males. Seventy-five participants (41 females) performed both a haptic and a MR task. Findings revealed a correlation between haptic processing and MR ability; specifically, higher performance in one predicted higher performance in the other. Importantly, this link was significant only when the haptic task was performed with the left hand, highlighting the role of the right hemisphere. Additionally, males outperformed females in both tasks, with the male advantage in haptic processing observed exclusively with left-hand use. These results suggest that the male advantage in haptic processing might contribute to the observed male advantage in MR ability. Overall, the findings support the notion that the sensorimotor system, influenced by hemispheric specialization, plays a role in shaping cognitive functions.

## Highlights

- The study of the relationship between the haptic-sensorimotor system and mental rotation ability has remained scarce.
- Studies have not examined how sex and hand performance contribute to the haptic and mental rotation relationship.
- Results showed that sex and hand performance were important factors influencing the relationship.
- The sensorimotor system may contribute to the sex differences in mental rotation ability.
- The sensorimotor system appears to help shape cognitive functions and their hemispheric lateralization.

## Introduction

The embodied cognition theory suggests that sensorimotor experiences shape human perception and interaction with the world [1–5]. Cognitive processes develop through early sensory and motor interactions, with evidence showing that the same sensorimotor systems used for physical actions are also involved in mental tasks like retrieving information [6, 7]. For instance, mental rotation—a cognitive ability to manipulate objects in the mind—activates brain areas like the premotor and primary motor cortices, which are responsible for planning and executing movements [8, 9]. As Kolb and Whishaw [10] noted, "mental manipulation is an elaboration of the neural control of actual manipulation." Exploring the link between the sensorimotor system and cognition is vital for understanding brain development and individual differences in cognitive abilities.

### Mental rotation and the haptic sensorimotor system

Research has broadly explored the link between mental rotation and the motor system, suggesting shared mechanisms [11–14]. For example, motor areas activate during mental rotation [9], and participants perform faster and more accurately when motor and mental rotation directions align [12]. Objects harder to physically rotate are also harder to mentally rotate [15], and embodied objects (e.g., gloves) are rotated faster than non-embodied ones (e.g., houses) [16]. Despite this evidence, little is known about the link between mental rotation and the haptic system, which integrates touch (cutaneous input) and proprioception (kinesthetic input) [17]. The haptic system is crucial for daily interactions, particularly in low-visibility situations (e.g., finding keys in a bag). Some studies suggest individuals with better mental rotation skills perform better on haptic tasks [18, 19]. Research in infants shows that manual exploration is key to developing mental rotation ability [20, 21], highlighting the role of the haptic system in cognitive development. The first goal of the current study was to further investigate the relationship between haptic perception and mental rotation.

### Mental rotation and the haptic system-sex differences

Mental rotation ability exhibits significant and consistent sex differences, with males generally outperforming females, particularly on the Mental Rotation Test (MRT, [22]). This pattern has been observed across the lifespan and in over 50 nations [23–28]. In contrast, research on sex differences in the haptic system is limited and inconclusive [29]. Some evidence suggests a male advantage: in haptic parallelity tasks, blindfolded males made smaller alignment errors than females [30–32], and males outperformed females in a shape–texture similarity judgment task [33]. However, other studies found a female advantage, such as in identifying changes in the positions of raised line pictures [34]. Additionally, some research has reported no sex differences, such as in a haptic version of the water level test [35, 36]. These findings highlight the scarcity and inconsistency in research on sex differences in haptic perception. The second goal of this study was to investigate sex differences in haptic perception and mental rotation ability.

### Mental rotation and the haptic system – hemispheric specialization

Research has long shown right hemisphere specialization for mental rotation [37, 38]. Studies using the Shepard & Metzler [22] task reveal a left visual field advantage in accuracy and response time in neurologically intact participants [39] and similar findings in commissurotomized patients and those with right (but not left) parietal lobe lesions [40]. Imaging studies pinpoint the right posterior parietal cortex as a key neural substrate for mental rotation, with consistent activity observed in the right hemisphere in meta-analyses [9, 41].

The right hemisphere also specializes in haptic perception. Patients with right hemisphere damage show greater impairment in haptic tasks, such as the Form Board Test, compared to those with left hemisphere damage [42]. Commissurotomized patients exhibit a left-hand/right-hemisphere advantage in tasks requiring organization of scrambled objects by shape or texture [43]. Other studies support this advantage in texture discrimination, tactual maze navigation, and shape recognition [44, 45]. Infants as young as four months old also show a preference for left-hand exploration [46]. Imaging studies further confirm that haptic shape processing is lateralized to the right posterior parietal lobule [47, 48]. These findings suggest shared neural substrates for haptic processing and mental rotation. The third aim of this study was to examine hand differences in haptic perception and their relationship to mental rotation ability.

To summarize, this study aimed to explore the relationship between haptic perception and mental rotation ability, with a particular focus on how sex and the hand used during haptic manipulation may influence this relationship. We hypothesized, first, a positive relationship between haptic processing and mental rotation ability; second, better performance by males in both functions; and third, a left-hand advantage in haptic processing with a stronger positive relationship between left-hand haptic performance and mental rotation ability.

## Methods

### Participants

Seventy-five (n = 41 females) healthy right-handed participants between the ages of 13 to 25 years old took part on this study. Participants self-reported their handedness, sex, and gender (in our sample, all participants were cisgender). Participants were recruited through word-of-mouth, media advertisements, and through the Department of Psychology at the University of Lethbridge, using participant management software (Sona Systems). Those that were university students, received course credits for their participation. The experiment was approved by the University of Lethbridge Human Subject Research Committee.

### Tasks

**Fig. 1:**
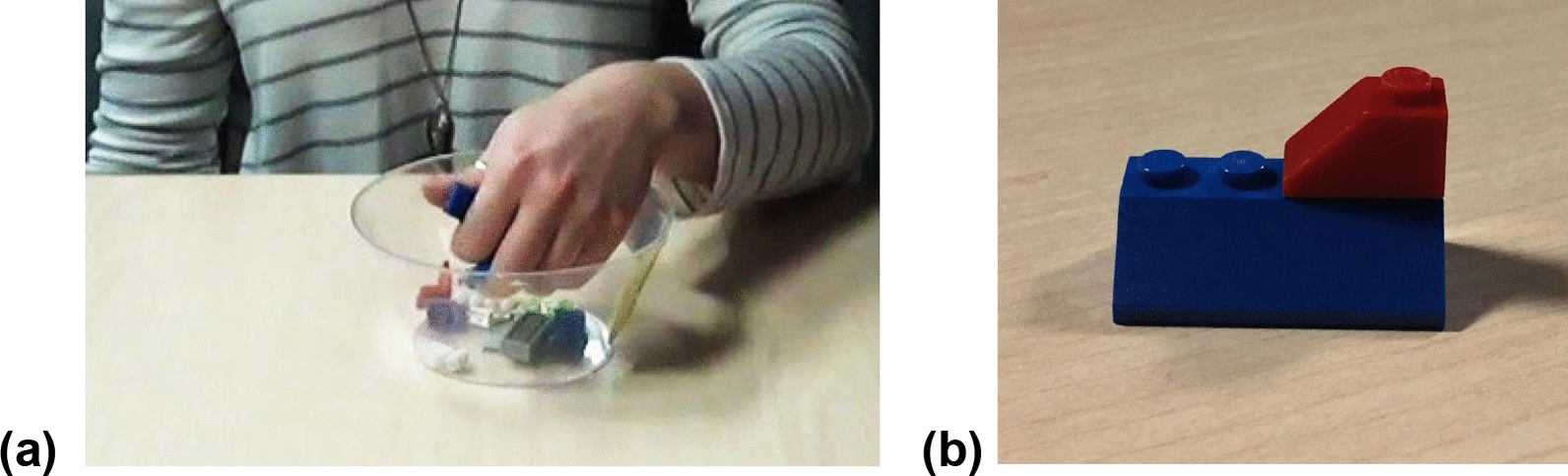
The Haptic Task**.** **a** The set up- participant picking from a bowl with 12 distinct Lego blocks, the two that they thought made up the model. **b** One 2-piece LEGO model

**Fig. 2:**
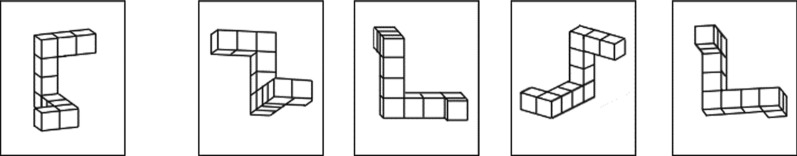
The Mental Rotation Test. One trial of the Mental Rotation Test (MRT). The target figure on the left was compared to the four figures on the right. The participant attempted to identify the two figures that were rotated versions of the target. The second and third figures matched the target figure in this example

Participants were given the haptic task (Fig. 1) and the MRT (Fig. 2). The haptic task consisted of a total of 12 trials, six trials were done with the left-hand and six trials with the right- hand. The order of the trials was randomized within participants. The order of the starting hand (left or right) was also counterbalanced between participants. Each trial consisted of blindfolded participant’s haptically exploring a simple 2-piece LEGO model (Fig. 1) for eight seconds with one hand. The Lego model was made out of two distinct pieces. Each of the 12 models were different. Immediately after the haptic exploration, the two pieces were placed in a bowl that contained 10 other unique pieces. The participant’s job was to haptically search inside the bowl for the two LEGO pieces that the made up the model they had just explored. To be clear, inside the bowl, the two pieces that made up the model were among twelve pieces; thus, ten pieces were distractors. The MRT consisted of two sets of 12 trials. The stimuli for each trial comprised a target figure on the left and four figures on the right (Fig. 2). The participant’s task was to identify the two figures that matched the target.

### Procedure

Participants were first asked to read and sign a consent form. Then participants were seated at a table (Fig. 1). For the haptic task participants were told they would be blindfolded, that they would feel a model with two LEGO pieces for a few seconds; they were then instructed to search for the two pieces that made up the model. They were told that they were only allowed to use one hand (either left or right) to feel the model and find the pieces, and that they could not use both hands. Furthermore, they were told that once they found the pieces, they would take them out of the bowl and locate them on the side, on top of the table. They were asked to do the task as accurate and as fast as they could. The experimenter then handed the participant a blindfold to cover their eyes. The bowl containing the twelve pieces was placed on top of the table, in front of the participant. The experimenter then placed the model (two pieces together) on the participants’ corresponding hand. The participants felt the model for eight seconds, starting when the experimenter placed the model on the participants’ hand and ending when the experimenter said the time was up. The experimenter then placed the two pieces inside the bowl containing the 10 distractors and started the stopwatch as soon as the participant began searching for the pieces inside the bowl. The experimenter stopped the stopwatch once the participant had placed the two pieces outside of the bowl and on the table. Each of the trials followed the same procedure. Following the haptic task, the MRT was given to the participants (Fig. 2). For the MRT, participants were instructed to choose the two stimuli out of the four options that matched the target stimuli. Participants were given a 3-min limit to complete each of the two sets with a 3-min break between sets (Peters et al. 1995).

### Data analysis

For the haptic task, there were two dependent variables: errors and time to complete the task. An error was recorded if the participant took out of the bowl a piece that did not match any of the two pieces that made up the model. The time taken to find the pieces inside each of the bowls (i.e. each trial) was recorded. The MRT was scored by taking the sum of only the trials in which the two answers were correct, dividing by the maximum score of 24, and then multiplying by 100.

Pearson correlation coefficients were calculated between the haptic task time, haptic errors, and MRT to examine the relationship between the haptic task and MRT performance. A mixed design ANOVA was used to investigate sex differences in the haptic task, and the MRT with Sex as fixed factor. Furthermore, to explore Sex and Hand differences in haptic task performance, a repeated measures ANOVA was used, with Sex as the between-participant factor and hand as within. Lastly, to investigate hand differences in haptic perception and its relationship to mental rotation ability, a linear regression analysis was conducted to determine if any of the dependent variables (left-hand errors, right-hand errors, left-hand time, and right-hand time), was the predictor of performance in the MRT. IBM SPSS Statistics (Version 29) was used for all analyses. The alpha level for all comparisons was 0.05.

## Results

### Haptic processing and MRT relationship

**Fig. 3:**
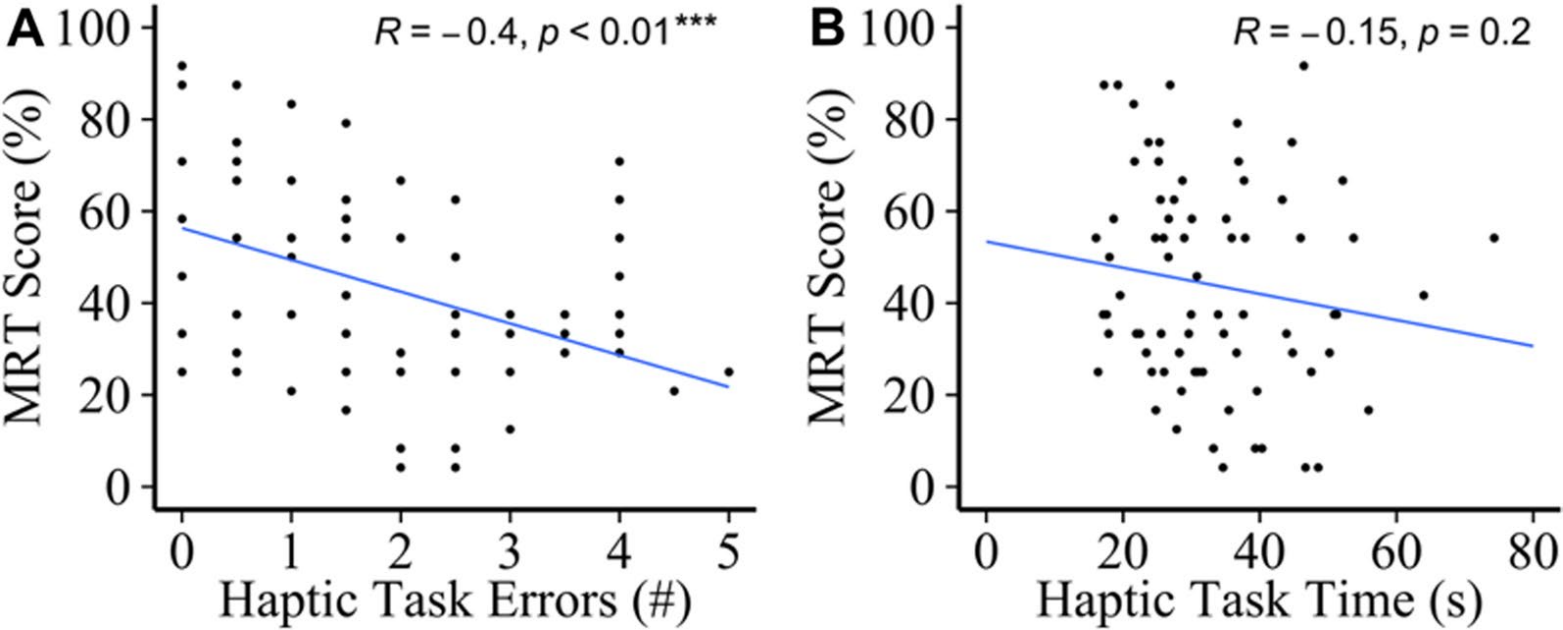
Results of the correlation analysis for haptic task and MRT performance

Figure 3 shows the results of the correlational analysis. There was a significant negative correlation (r = − 0.40) between the number of errors on the haptic task and MRT performance, the more errors the participants made, the worse their performance on the MRT. No significant difference was found for the amount of time participants took in solving the haptic task.

### Sex differences—haptic task and MRT

The mixed design ANOVA (see Table 1) revealed a significant main effect of Sex in the number of errors F _(1,73)_ = 12.19, p < 0.001, η^2^_p_ = 0.14 and in the time F _(1,73)_ = 7.62, p < 0.01, η^2^_p_ = 0.09 participants took in the haptic task. Males made fewer errors than females and took less time than females but this was only true for the left-hand. There was not a significant effect of Sex in the errors or time participants took in the haptic task with their right hand. For the MRT task, a significant main effect of Sex, F _(1,73)_ = 9.87, p < 0.01, η^2^_p_ = 0.12 was found, males had better scores than females (Table 1).

**Table 1:** Means and standard errors for the dependent variables. Please note the sex difference when using the left, but not the right hand

Dependent variable	Grand mean	Female participants	Male participants	*F* statistic
Haptic Left Errors (#)	1.76 ± 0.20	2.34 ± 0.29	1.06 ± 0.21	F _(1,73)_ = 12.19, p < 0.01, η^2^_p_ = 0.14
Haptic Left Time (s)	31.99 ± 1.49	35.58 ± 2.18	27.65 ± 1.75	F _(1,73)_ = 7.62, p < 0.01, η^2^_p_ = 0.09
Haptic Right Errors (#)	1.8 ± 0.18	2.00 ± 0.26	1.60 ± 0.26	F _(1,73)_ = 1.34, p > 0.1, η^2^_p_ = 0.18
Haptic Right Time (s)	32.97 ± 1.56	34.18 ± 2.13	31.51 ± 2.31	F _(1,73)_ = 0.72, p > 0.1, η^2^_p_ = 0.10
MRT score (%)	43.94 ± 2.61	38.89 ± 3.20	52.45 ± 3.83	F _(1,73)_ = 9.87, p < 0.01, η^2^_p_ = 0.12

### Haptic task- sex and hand differences

**Fig. 4:**
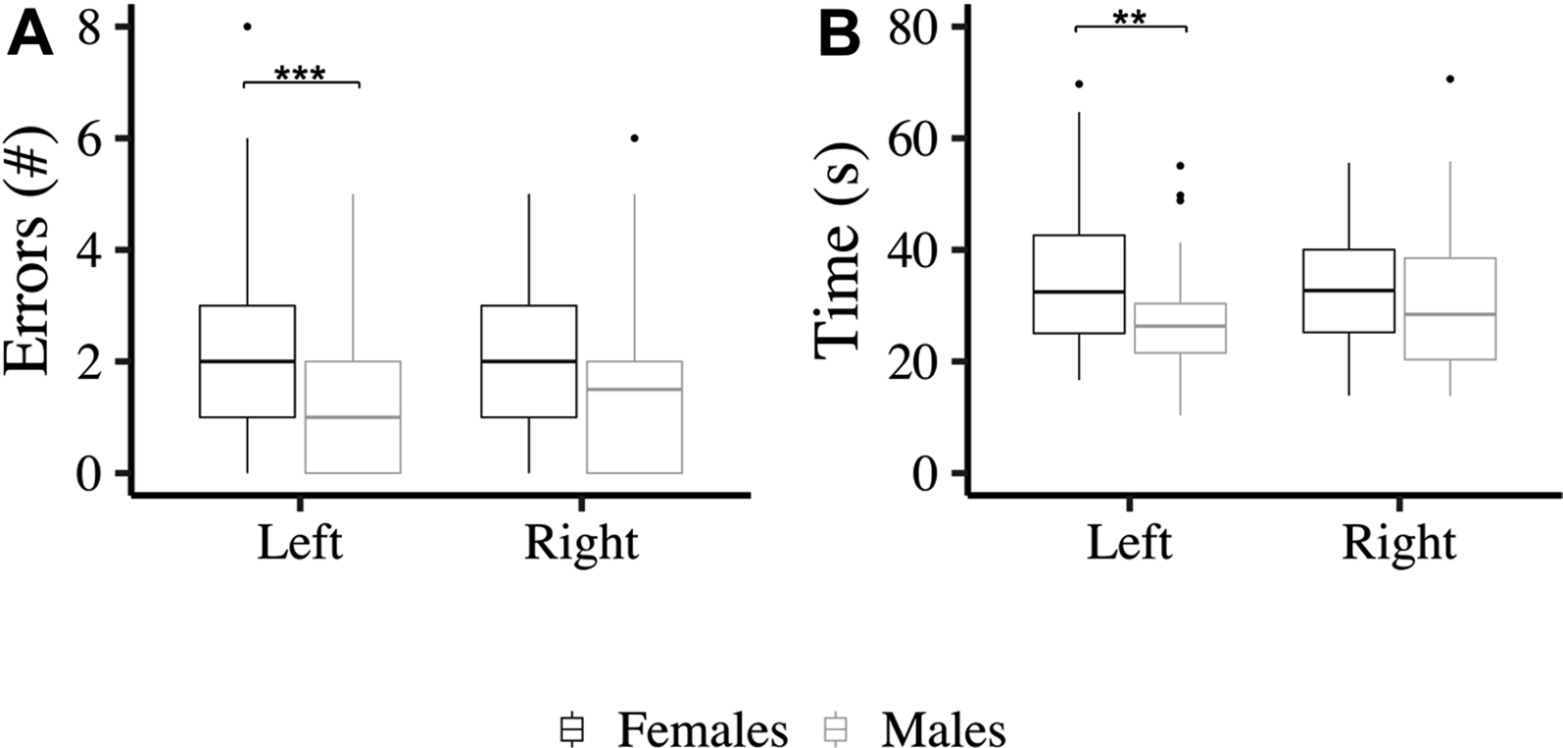
Haptic Task errors and times for each Hand (left, right) and Sex (female, male). p ≤ 0.01**, p ≤ 0.001***. Error bars represent standard errors

Performance on the haptic task was analyzed with Sex (female, male) by Hand (left, right) repeated measures ANOVA, with Sex as between-participant factor and Hand as a within-participant factor. Regarding haptic task errors, a significant main effect of Sex was found F _(1,73)_ = 8.49, p < 0.01, η^2^_p_ = 0.07 and a significant Sex by Hand interaction F _(1,73)_ = 4.09, p < 0.05, η^2^_p_ = 0.02. Males made fewer errors than females when using their left-hand but not when using their right hand (Fig. 4A). Regarding haptic task times, a significant main effect of Sex was found F _(1,73)_ = 4.09, p < 0.05, η^2^_p_ = 0.04; with males completing the task faster. A Sex by Hand interaction approached significance F _(1,73)_ = 3.16, p = 0.08, η^2^_p_ = 0.01. To further explore this marginal interaction, t-tests were conducted between male and female participants’ performance with the right and left-hands. Males were significantly faster than females when using their left-hand but not when using their right hand (Fig. 4B).

### Haptic task and MRT relationship—hand differences

To further explore the relationship between left- and right-haptic task performance and mental rotation ability, a regression analysis was used. The model included MRT as the dependent variable and left-hand haptic task errors, right-hand haptic task errors, left-hand haptic time, and right-hand haptic time as potential predictors (see Table 2). The multiple linear regression analysis was significant (F _(1,73)_ = 4.49, p < 0.01, R^2^ = 0.20). The left-hand number of errors was the sole significant predictor of MRT performance (p < 0.01).

**Table 2:** Results of the regression analysis

Independent variables	Unstandardized *B*	Coefficients of standard error	Standardized coefficients beta	t	Sig.
(Constant)	65.69	8.01		8.16	< 0.01
**Left-hand errors (#)**	**− 4.43**	**1.55**	**− 0.33**	**− 2.86**	**0.01**
Right-hand errors (#)	− 2.22	1.69	− 0.15	− 1.32	0.19
Left-hand time (s)	− 0.22	0.23	− 0.13	− 0.99	0.33
Right-hand time (s)	− 0.08	0.21	− 0.05	− 0.40	0.69

The only significant predictor of MRT performance was the left-hand errors

## Discussion

The results of the current investigation revealed a significant relationship between errors in the haptic task and performance on the Mental Rotation Test (MRT): more errors in the haptic task were associated with poorer MRT performance, supporting our first hypothesis. This finding aligns with the embodied cognition theory, which posits that active physical manipulation of objects is linked to mental manipulation. Shepard [49] proposed that mental representations are “isomorphic” to physical rotation, and previous studies have shown that physical rotation can hinder or enhance mental rotation depending on directional alignment [50, 51]. However, these studies primarily involved tactile stimulation or handheld sensors, whereas the current study’s haptic task included active object manipulation, engaging both touch and proprioception, making it more ecologically valid.

Interestingly, no significant relationship was found between haptic task completion time and MRT performance, although the correlation was in the expected direction (longer times were associated with worse MRT scores). This lack of significance may be due to high variability in task completion times; some participants may have quickly stumbled upon the target pieces, while others took longer, independent of their accuracy. Indeed, the number of errors was not correlated with task time (r = 0.02), suggesting accuracy and speed are not directly related in the haptic task. Previous research has also shown that speed and accuracy do not always align [52]. While speed measures can provide insights into cognitive processes, their relationship with accuracy is complex and sometimes inconsistent [53]. This discrepancy might stem from the dual-process theory, which distinguishes between fast, automatic thinking and slower, deliberate reasoning [54]. These two modes of thinking could explain the variability in how participants approached the haptic task and the MRT.

Regarding sex differences, results showed that males made fewer errors in the haptic task and scored better in the MRT. Thus, our second hypothesis was supported, a male advantage was found in both tasks. These results are in line with other researchers who have found a male advantage in haptic perception. These studies, however, have mostly used the haptic parallelity task [30–32, 55, 56] where participants aligned a reference bar (previously placed in a different orientation) to the test bar. The parallelity task taps mostly on the ability to orient objects in space and less on haptic discrimination of shape and/or texture (as the task used in the current study). To our knowledge few studies have explored sex differences in haptic discrimination of shape and/or texture [33, 57], with Cohen and Levy (1986) [33], reporting a male advantage. Thus, our findings make a significant contribution to the literature by showing a male advantage specifically for haptic processing of object shape. As well, our findings add to the large body of literature supporting a male advantage in mental rotation ability. To our knowledge, this is the first study to show a male advantage in these two processes in the same sample of participants.

When examining sex and hand performance in the haptic task, results showed that males outperformed females by making fewer errors and completing the task faster, but only when using the left hand. This aligns with Witelson’s [57] findings, where boys (ages 6–13) exhibited a left-hand advantage in a haptic shape discrimination task, though no overall sex differences were found. Witelson attributed this left-hand advantage to the right hemisphere’s specialization for spatial processing which, Witelson argued, is more pronounced in males, while females exhibit more bilateral processing. Subsequent studies (e.g., Nilsson & Geffen [58]) replicated the male left-hand advantage and similarly linked it to right hemisphere spatial strategies, contrasting with females’ likely use of bilateral cognitive strategies. This male bias toward right hemisphere dominance in spatial tasks, including mental rotation [59, 60], may explain the left-hand advantage observed in haptic tasks. Supporting this view, clinical and imaging studies suggest males show stronger hemispheric specialization, while females exhibit greater inter-hemispheric connectivity [61, 62]. However, other research has failed to replicate these findings (e.g., Cranney & Ashton [63]) or has reported a right-hand advantage with no sex differences [64]. These inconsistencies highlight the need for further research exploring the interplay between sex, hand dominance, and haptic processing to determine whether the left-hand male advantage stems from spatial processing, haptic strategies, or an interaction of these factors.

To more directly examine the relationship between hand performance on the haptic task and the MRT, a regression analysis was done. Results showed that the number of errors with the left-hand was the only predictor of MRT performance. Thus, confirming our hypothesis of a left-hand positive relationship with mental rotation. This finding expands previous literature of right hemisphere specialization for haptic processing and mental rotation ability [9, 48] to demonstrate that these processes are linked. Therefore, the results suggest that haptic processing, and mental rotation may share neural substrates which have been identified in the parietal cortex [65, 66]. However, behavioural experiments that include imaging techniques aimed at exploring this relationship are missing. Future research should first, explore if these processes share neural substates; second, if there is sexual dimorphism in these substrates; and third, how these brain processes (i.e., haptic and mental rotation) interact during development. Overall, research addressing these aspects could provide insights into the importance of the haptic system for the development of spatial cognition and the associated sex differences in these processes.

One limitation of this study lies in the specific nature of the task. Unlike previous research, which often employed nonsensical shapes or objects with minimal tactile cues [48, 54, 58] this study utilized Lego bricks, which may provide richer haptic information. Future research could explore a wider variety of objects with varying levels of haptic complexity to determine whether task difficulty modulates sex differences in haptic processing.

In conclusion, the results of this study support a close relationship between haptic processing and mental rotation ability that is influenced by sex and hand. A male advantage was found in haptic processing and mental rotation ability. However, the male advantage in haptic processing appears to exist only for the left hand. Furthermore, only a left-hand relationship was found with MRT performance. In sum, this study provides evidence of how the sensorimotor system and cognition are interrelated, noting that this interrelatedness differs by sex.

## Data Availability

The materials and data that support the findings of this study are available from the corresponding author upon reasonable request.
